# Effect of explant type (leaf, stem) and 2,4-D concentration on callus induction: influence of elicitor type (biotic, abiotic), elicitor concentration and elicitation time on biomass growth rate and costunolide biosynthesis in gazania (*Gazania rigens*) cell suspension cultures

**DOI:** 10.1186/s40643-022-00588-2

**Published:** 2022-09-16

**Authors:** Huda E. Mahood, Virginia Sarropoulou, Thiresia-Teresa Tzatzani

**Affiliations:** 1 Department of Horticulture, College of Agriculture, University of Al-Qadisiyah, Al Diwaniyah, 58002 Iraq; 2 Institute of Plant Breeding and Genetic Resources, Laboratory of Protection and Evaluation of Native and Floriculture Species, Hellenic Agricultural Organization (HAO)-DEMETER, Balkan Botanic Garden of Kroussia, Thermi, P.O. Box 60458, P.C. 570 01 Thessaloniki, Greece; 3 Institute of Olive Tree, Subtropical Crops & Viticulture, Laboratory of Subtropical Plants & Tissue Culture, Hellenic Agricultural Organization (HAO)-DEMETER, 167 K. Karamanlis Avenue, 73134 Chania, Greece

**Keywords:** Biomass yield, Callus induction, Cell suspension cultures, Costunolide, Elicitors, Gazania, Medicinal plants, Plant tissue culture, Secondary metabolites, Sesquiterpene lactones

## Abstract

*Gazania rigens* (L.) Gaertn. (Asteraceae) is a medicinal plant with high ornamental potential and use in landscaping. The therapeutic potential of sesquiterpene lactones (SLs) as plant natural products for pharmaceutical development has gained extensive interest with costunolide (chemical name: 6E,10E,11aR-6,10-dimethyl-3-methylidene-3a,4,5,8,9,11a-hexahydrocyclodeca[b]furan-2-one) used as a popular herbal remedy due to its anti-cancer, antioxidant, anti-inflammatory, anti-microbial, anti-allergic, and anti-diabetic activities, among others. In the present study, two explant types (leaf, stem) and four 2,4-dichlorophenoxy acetic acid (2,4-D) concentrations (0, 0.5, 1 and 2 mg/L) were tested for callusing potential. The results showed that stem explants treated with 1.5 mg/L 2,4-D exhibited higher callus induction percentage (90%) followed by leaf explants (80%) with 1 mg/L 2,4-D, after a 4-week period. Cell suspension cultures were established from friable callus obtained from stem explants following a sigmoid pattern of growth curve with a maximum fresh weight at 20 days of subculture and a minimum one at 5 days of subculture. In the following stage, the effects of elicitation of cell suspension cultures with either yeast extract (YE) or methyl jasmonate (MeJA), each applied in five concentrations (0, 100, 150, 200 and 250 mg/L) on cell growth (fresh and dry biomass) and costunolide accumulation were tested. After 20 days of culture, YE or MeJA suppressed cell growth as compared to the non-elicited cells, while costunolide accumulation was better enhanced under the effect of 150 mg/L MeJA followed by 200 mg/L YE, respectively. In the subsequent experiment conducted, the optimal concentration of the two elicitors (200 mg/L YE, 150 mg/L MeJA) was selected to investigate further elicitation time (0, 5, 10, 15 and 20 days). The results revealed that YE biotic elicitation stimulated cell growth and costunolide production, being maximum on day 20 for fresh biomass, on day 5 for dry biomass and on day 15 for the bioactive compound. Accordingly, cell growth parameters were maximized under the effect of abiotic elicitation with MeJA for 15 days, while highest costunolide content was achieved after 10 days. Overall, MeJA served as a better elicitor type than YE for biomass and costunolide production. Irrespective of elicitor type, elicitor concentration and elicitation time, maximal response was obtained with 150 mg/L MeJA for 10 days regarding costunolide accumulation (18.47 ppm) and 15 days for cell growth (fresh weight: 954 mg and dry weight: 76.3 mg). The application of elicitors can lead the large quantity of costunolide to encounter extensive range demand through marketable production without endangering of *G. rigens*.

**Graphical Abstract:**

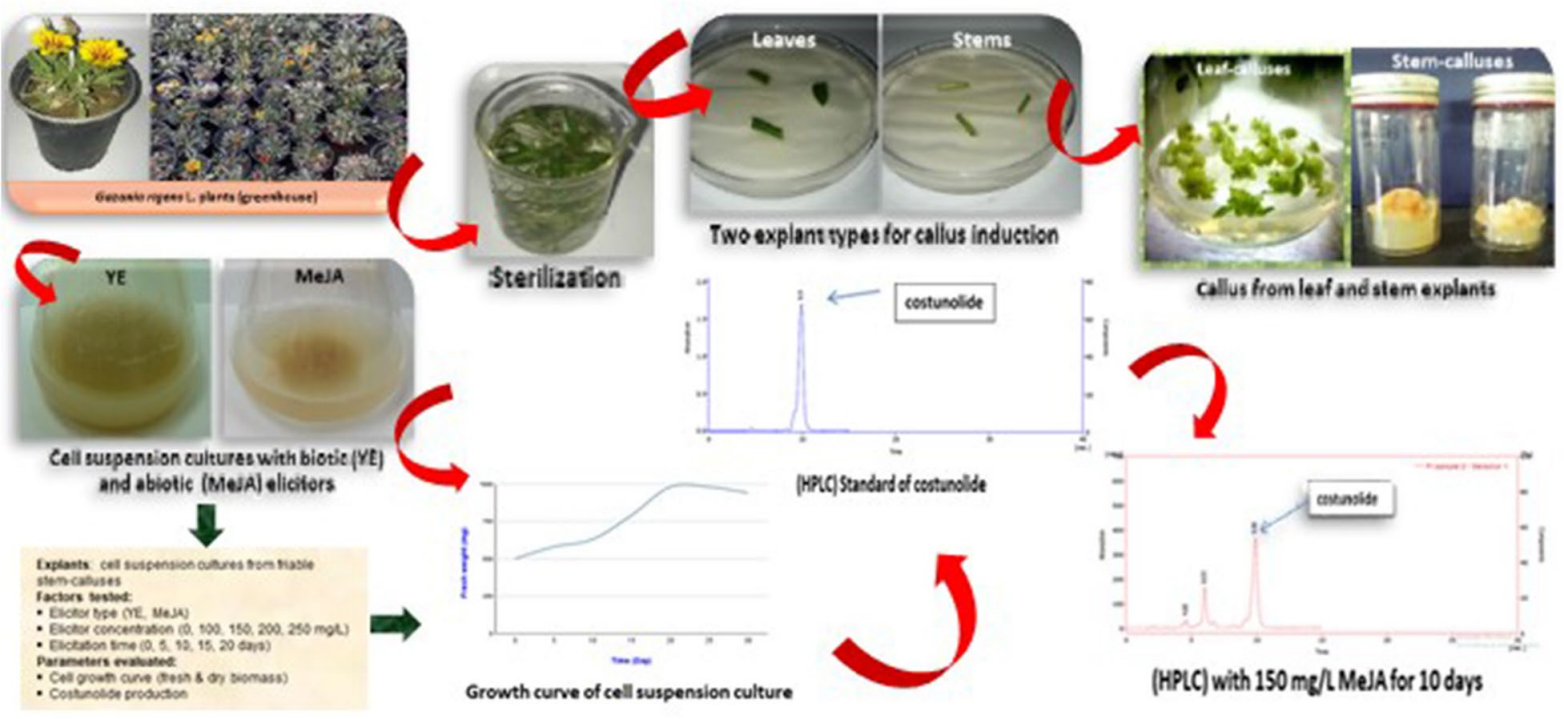

## Introduction

*Gazania rigens* (L.) Gaertn. (Asteraceae family) is a herbaceous perennial plant, native to South Africa that grows better in warm and sunny locations (Xie et al. 2013). *Gazania rigens* plants have colorful flowers, a prolonged flower life and flowering season, are resistant to adverse environmental conditions, such as drought, heat, and moderate cold temperatures, and well-adapted to poor soils (Li 2011). *Gazania rigens* is easily propagated by cuttings, plant division, and tissue culture and presents high ability of creating colonies in roadbed landscapes (Wang 2013).

**Fig. 1:**
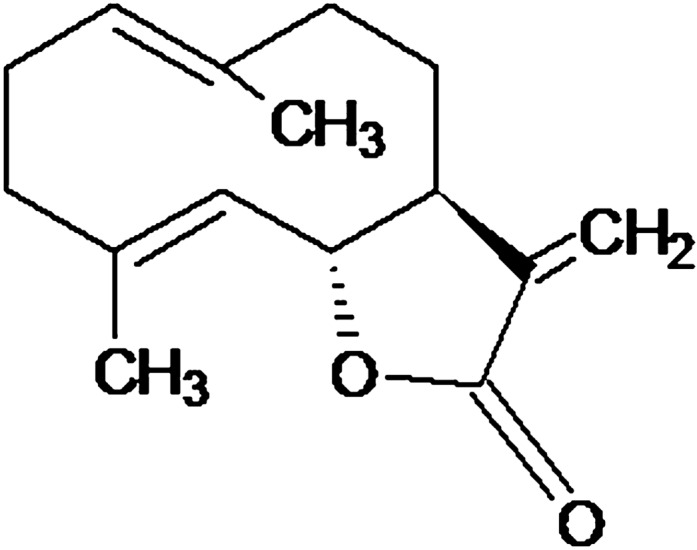
Chemical structure of costunolide (Rasul et al. 2012) as the target secondary metabolite

Many studies have pointed out the beneficial effects and the biological properties of the essential oils and their major components including terpenes and terpenoids (mostly monoterpenes and sesquiterpenes) (Stephane and Jules 2020). The therapeutic potential of sesquiterpene lactones (SLs) as plant natural products for pharmaceutical development has gained extensive interest and investigated thoroughly the recent past years (Muschietti and Ulloa 2016). The majority of SLs have been reported from the Asteraceae family (de Kraker et al. 2002); however, there are variations in their structure and their backbones are constrained to a limited set of core skeletons, such as germacranolide, eudesmanolide and guaianolide (Van Beek et al. 1990), where costunolide is the common precursor (de Kraker et al. 2002). Costunolide, a well-known SLs is used as a popular herbal remedy due to its anti-cancer activities (Rasul et al. 2012) with numerous other therapeutic effects including antioxidant, anti-inflammatory, anti-microbial, anti-allergic, anti-diabetic, bone remodeling, prevention of neurodegenerative disease, inhibition of alopecia, and prevention of lung disease (Kim and Choi 2019). Costunolide (6E,10E,11aR-6,10-dimethyl-3-methylidene-3a,4,5,8,9,11a-hexahydrocyclodeca[b]furan-2-one) is a colorless crystalline powder with molecular formula of C_15_H_20_O_2_ and molecular weight of 232.318 g/mol (Rasul et al. 2012). Structurally, costunolide (Fig. 1) is a monocarboxylic acid having three double bonds which by catalytic hydrogenation generates hexahydrocostunolide and partial hydrogenation of costunolide produces dihydrocostunolide (Rao et al. 1960). The bioactivity of costunolide is mediated through its functional moiety, α-methylene-γ-lactone, which can react with the cysteine sulfhydryl group of various proteins, thereby altering intracellular redox balance (Rasul et al. 2012).

Secondary metabolites (SMs) are necessary for maintaining plant life cycle and environmental adaptation reasons (Park et al. 2020). The increase in world population and the reduction in cultivable available land (Rao and Ravishankar 2002) along with environmental and geopolitical instabilities as well as rapid depletion of medicinal plants from their natural habitat are limiting factors in the production of plant-derived compounds (Mulabagal and Tsay 2004), because large-scale crop cultivation is needed for their extraction on a commercial basis (Kieran et al. 1997). A wide range of uses have been proposed for SMs but mainly as drugs, flavorings, fragrances, pigments, bio-pesticides, and food additives (Murthy et al. 2014). Plant genetics, environmental conditions, climate, season, growth period, plant parts, pre- and post-harvest processes, extraction methods (Açıkgöz 2020), cultivation practices (Nagella and Murthy 2011) as well as the age of the plant (Nasim et al. 2010) are among the factors that affect the biosynthesis of SMs, increasing the cost of their production (Rao and Ravishankar 2002). The in vitro culture of plant cells, tissues and organs has been associated with higher production of SMs than the field grown plants (Nasim et al. 2010). Plant tissue culture has been an effective and alternative method in the production of SMs because of its reliability and predictability as the effect of the main external factors (e.g., geographical, seasonal and environmental) is nullified, the unwanted taste can be altered or eliminated (Abd El-Salam et al. 2015), thus production rapidity of high quality and standard products are guaranteed (Rao and Ravishankar 2002). Cell suspension culture is the best in vitro plant tissue culture system to fulfill the ever escalating industrial demand for increased production of SMs (Rani et al. 2020), due to the fast growth rate of cells in suspension (Chan et al. 2010). Elicitation has been the most commonly used technique (easy, high efficiency, low expenses) (Murthy et al. 2014) for the successful production of SMs by triggering their biosynthetic pathway (Cai et al. 2012).

2,4-Dichlorophenoxy acetic acid (2,4-D) is a very effective plant growth regulator of the auxins group in stimulating the formation of calli cells and produces crumb or friable callus (Mahadi et al. 2016). The 2,4-D hormone also has more stable properties compared to the other types of auxin, because it is not easily decomposed by enzymes released by explants or by heating during the sterilization process (George et al. 2008). The friable callus is needed as a raw material for cell suspension so that the callus can be easily separated from each other so that it will be easy to be suspended into liquid media, into a single cell and then elicited (Damayanti et al. 2020). The exogenous application of elicitors (abiotic, biotic based on their nature or form) along with a plant membrane receptor is involved in the activation of specific genes, enhancing the accumulation of targeted SMs (Thiruvengadam et al. 2015). Yeast extract (YE) has been used as a biotic elicitor, while plant growth regulators, such as salicylic acid, jasmonic acid, and methyl jasmonate (MeJA) as abiotic elicitors (Baenas et al. 2014). SMs biosynthensis in cell or hair root cultures have been strengthened after elicitation with YE and MeJA (Krstić-Milošević et al. 2017). YE, rich in vitamins of the B-complex and other essential components such as chitin, *N*-acetyl-glucosamine oligomers, β-glucan, glycopeptides and ergosterol (Boller, 1995) is actively participates in the propulsion of the metabolites synthesis and initiation of plant defense responses (Cai et al. 2012). MeJA acts as a signaling molecule in the phenylpropanoid pathway by triggering the effective stress response (Thiruvengadam et al. 2015; Ho et al. 2018). An efficacious tool to augment parthenolide (PN) (a sesquiterpene lactone compound) production could be the use of elicitors such as YE and MeJA due to their non-destructive nature for plant tissues related to terpene accumulation (Majdi et al. 2011).

The previous studies about in vitro culture of gazania have focused on the production of plants only during the common micropropagation culture stages. Therefore, this study was carried out to establish an efficient callus regeneration protocol of the *G. rigens* plant, using different explant types (leaves, stems) cultured in liquid nutrient medium supplemented with different 2,4-D concentrations. Besides, the study also aimed to quantify biomass yield production and total costunolide content of cell suspension cultures derived in vitro from stem-calluses under the effect of different elicitor types (yeast extract, methyl jasmonate), elicitor concentrations and elicitation exposure times. To the best of our knowledge, there is no report for elicitation in the genus *Gazania*; therefore, the original aspect of the present work is that for the first time an elicitor-enhanced metabolites production in *G. rigens* is reported.

## Materials and methods

### Plant material and sterilization

The garden of Diwaniya city in Iraq provided 2-month-old Gazania plantlets grown in a greenhouse. Leaf and stem explants were surface sterilized with 70% (v/v) ethanol for 30 s and three times washed with sterile distilled water, then 10 min in 5% (v/v) of sodium hypochlorite solution (containing 5.25% of Cl_2_) and three times washed with sterile water. The explants were placed in 250 mL flask with 50 mL of Murashige and Skoog medium (MS) (Murashige and Skoog 1962) supplemented with 30 g/L sucrose, 8 g/L agar, and 2,4-D as a plant growth regulator in different concentrations (0, 0.5, 1, and 1.5 mg/L).

### Callus induction

For callus induction, a two-factor multifactorial design was employed. The first factor was the explant type, which was separated into two categories: leaf explants and stem explants. The second factor was 2,4-D, which had four levels: 0, 0.5, 1, and 1.5 mg/L. The explants were preserved in the previous section's media and incubated at 25 °C with a 16-h photoperiod. Each treatment was replicated three times with ten explants per replicate (total 30 explants per treatment). As a control, the explants were cultured without plant growth regulators. After 4 weeks, percentage of callus formation (%) was recorded.

### Establishment of cell suspension cultures

*Gazania rigens* cell suspension cultures were established from friable callus obtained from stem explants. A passage of calli (500 mg) was re-cultured into a 120 mL Erlenmeyer flask containing 25 ml of MS liquid culture medium supplemented with 1.5 mg/L of 2,4-D. Then cultures were incubated on a rotary shaker (110 rpm) at 25 ± 2 °C under a photoperiod of 16 h/8 h (light/dark) at a light intensity of 1000 lx. Cell suspension cultures were sub-cultured at 2-week intervals. To maintain the cell cultures, the experiments were carried out in MS liquid culture medium supplemented with the same concentration of 2,4-D, pH, 5.8. After 5, 10, 15, 20, 25 and 30 days of culture, fresh cell weight was measured as described by Farjaminezhad and Garoosi (2021). For this purpose, the cells were collected by Whatman No. 1 filter paper using Büchner funnel under vacuum conditions for 30 s and weighed immediately.

### Treatment with elicitors

YE and MeJA used as biotic and abiotic elicitors, respectively. The cell suspension cultures were transferred into 100 mL Erlenmeyer flasks containing 25 mL of liquid MS medium supplemented with 1.5 mg/L of 2,4-D with an initial callus of 500 mg. The stock solution of YE (Merck, Germany) was prepared by dissolving yeast extract in distilled water and then filtering it using a 0.22 μm syringe filter. Different concentrations of YE including 0, 100, 150, 200 and 250 mg/L, or different concentration of MeJA including 0, 100, 150, 200 and 250 mg/L were added to cell suspension cultures. The cultures were kept on a rotary shaker at 110 rpm and 25 ± 2 °C in the dark, and sampling was done by recording fresh and dry weights after 20 days of each treatment depending on the previous results. Optimal concentration of elicitors was selected to investigate elicitation time (5–20 days). The addition of elicitors at the beginning of cell culture was used as the control. The cells were also harvested after 20 days of culture to evaluate the growth and costunolide accumulation.

### Preparation of sample solution from extracts

The ethanolic extracts of callus and cell suspension were obtained according to the method of Tshabalala et al. (2016). Callus and cell suspension from three replicates of each treatment were undergo drying at 40 °C to constant weights in an oven. They were subsequently pulverized into a fine powder using Waring Commercial Laboratory electric blender and stored at 4 °C. The powdered sample of 50 g was extracted with 500 mL of ethanol and in lidded 2 L flasks at 110 rpm for 24 h were shaken at 25 °C using an orbital shaker. The resulting infusion was filtered and evaporated to dryness in a rotary evaporator (Cole Parmer SB 1100, Shangai, China) and stored as dry extract at 20 °C until use. The individual concentrated extracts were diluted with methanol and injected onto HPLC system for the estimation of costunolide.

### Preparation of standard solution and quantification of costunolide content

Accurately weighed amount of 10 mg of costunolide was dissolved in 10 mL methanol in volumetric flask. Calibration standards were prepared by diluting the appropriate volume of stock solution with methanol to obtained concentration levels of 1, 2, 5, 10, 20, 50 and 100 μg/mL. Samples analyses were carried out on a HPLC system consisted of a Waters 600 HPLC with a 486 UV detector and 717 Autosampler. Chromatographic separation was performed on a Thermo BDS HYPERSIL C18 column (4.6 mm × 100 mm, 2.4 μm). The mobile phase was delivered at a flow rate of 0.5 mL/min consisting methanol–water solution (70:30 v/v). The column temperature was maintained at 25 °C and the effluent was monitored at 225 nm.

### Statistical analysis

Analysis of variance (ANOVA) was performed with the SPSS 17.0 statistical package and mean separation with Duncan's Multiple Range Test. Significance was recorded at *p* ≤ 0.05. The experimental layout was completely randomized.

The callus induction experiment was a 2 × 4 factorial one with two types of explants (stem, leaf) and four concentrations of 2,4-D (0, 0.5, 1.0, 1.5 mg/L), thus included eight treatments each replicated three times with 10 explants per replicate (30 explants/treatment). The main effect of factors (2,4-D concentration, explant type) and their interaction was determined by General Linear Model/two-way ANOVA. In addition, one-way ANOVA used for the comparison of means derived from the four 2,4-D concentrations for each explant type separately.

In the experiment related to the effect of elicitors on biomass cell growth parameters and costunolide accumulation, the means were subjected to one-way ANOVA for each elicitor type separately, regardless of elicitor’s concentration. In addition, the main effect of factors; elicitor type, elicitor concentration and their interaction was determined by General Linear Model/two-way ANOVA. The experiment was a 2 × 5 factorial one with two elicitors types (YE, MeJA) each applied at five concentrations (0, 50, 100, 150, 200 mg/L), thus consisted of 10 treatments (3 replicates × 10 explants/replicate = 30 explants/treatment).

In the experiment regarding the effect of elicitation time combined with either 200 mg/L YE or 150 mg/L MeJA on cell growth biomass and costunolide accumulation, the means were subjected to one-way ANOVA for each elicitor type separately, regardless of elicitation time. In addition, the main effect of factors; elicitor type, elicitation time and their interaction was determined by General Linear Model/two-way ANOVA. The experiment was a 2 × 5 factorial one with two elicitors types (YE, MeJA) and five elicitation periods (0, 5, 10, 15, and 20 days), thus 10 treatments (3 replicates × 10 explants/replicate = 30 explants/treatment).

## Results

### Explant sterilization and callus induction

Leaf- and stem-explants had different responses in sterilization percentage. The highest sterilization % were obtained in leaves with 90% and stems with 80%. However, the survival percentage of the explants after sterilization was 95% for leaf- and 90% for stem explants.

In the case of leaf explants as a single factor, 2,4-D gave similar callus induction percentages (70–80%) to the control (60%) without a significant difference (*p* = 0.330 > 0.05). As concerns stem explants, callus induction was considerably stimulated (80–90%) with 1 and 2 mg/L 2,4-D, in relation to the control (50%) (*p* = 0.013 < 0.05) (one-way ANOVA) (Table 1).

**Table 1:** Effect of explant type and 2,4-D concentration on callus induction (%) after 4 weeks of growth

Origin of callus	2,4-D (mg/L)	Callus induction (%)
Stem	0.0	50.0 ± 5.8^b(C)^
Stem	0.5	70.0 ± 0.0^ab(B)^
Stem	1.0	80.0 ± 10.0^a(AB)^
Stem	1.5	90.0 ± 5.8^a(A)^
*p* values (one-way ANOVA) (stem explants, 2,4-D concentration)		0.013*
Leaf	0.0	60.0 ± 5.8^a(BC)^
Leaf	0.5	70.0 ± 5.8^a(B)^
Leaf	1.0	80.0 ± 5.8^a(AB)^
Leaf	1.5	70.0 ± 10.0^a(B)^
*p* values (one-way ANOVA) (leaf explants, 2,4-D concentration)		*0.330 ns*
*p *values (two-way ANOVA/general linear model)		
2,4-D concentration (A)		0.006**
Explant type (B)		0.045*
(A)*(B)		0.020*

Means (*n* = 30) ± standard error (S.E.) with the same letter in a column are not statistically significant different from each other according to the Duncan’s multiple range test at *p* ≤ 0.05. ns *p* > 0.05; *p ≤ 0.05; **p ≤ 0.01. Superscript small letters—differences between four 2,4-D concentrations for each explant type (either stem or leaf) separately (one-way ANOVA). Superscript capital letters in parenthesis—differences between samples from the combined effect of two explant types (leaf, stem) and four 2,4-D concentrations (two-way ANOVA)

Based on two-way-ANOVA and General Linear Model, the main effect of factors involved [2,4-D concentration (*p* = 0.006), and explant type (*p* = 0.045)] and their interaction (*p* = 0.020 < 0.05) had a significant effect on callus induction percentage. The increase in callus induction % was more pronounced in the case of stem explants (50–90%), whereas non-significant differences were recorded among treatments in the case of leaf explants (60–80%). Therefore, stems treated with 1.5 mg/L 2,4-D followed by leaves with 1.0 mg/L 2,4-D were the two treatments yielding higher callus induction, 90% and 80%, accordingly without significant difference (Table 1).

**Fig. 2:**
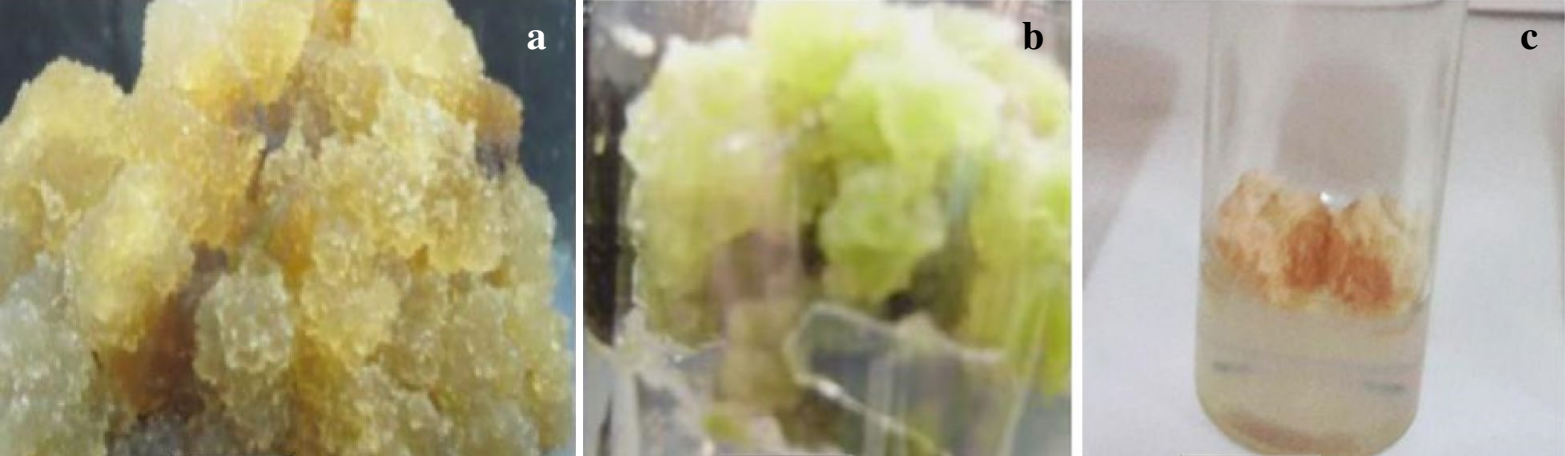
Callus obtained from *Gazania rigens* explants after 4 weeks of incubation: **a** callus from stem explant with 1.5 mg/L 2,4-D, **b** callus from leaf explant with 1.0 mg/L 2,4-D, and **c** callus from control treatment (2,4-D-free)

After 4 weeks of incubation and growth, the explant type, either leaf or stem, and 2,4-D concentration had an effect on the type and morphology of callus formation. The callus formed from the stem was friable, white–yellow colored and fast-growing (Fig. 2a), whereas the callus formed from the leaf was green, hard, and compact (Fig. 2b). Calluses obtained without 2,4-D were phenolizated (Fig. 2c), whereas calluses obtained with 2,4-D were no penalized areas (Fig. 2a, b). As a result, 2,4-D played a critical role in the induction of callus in gazania.

### Cell suspension cultures growth curve

**Fig. 3:**
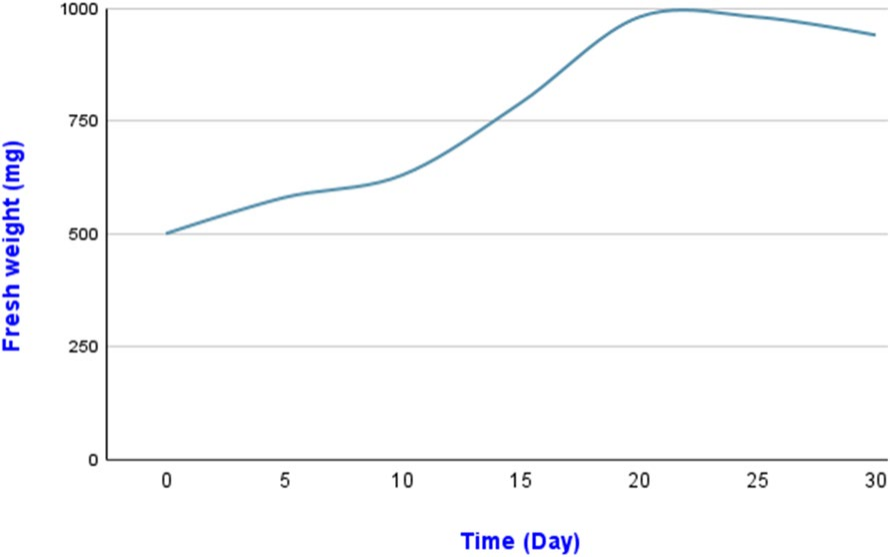
Cell suspension growth curve of *Gazania rigens* based on measurement of fresh weight for 30 days

During the first 5 days of cell suspension, fresh weight (mg) was recorded and started to grow for the subsequent 30 days of culture (Fig. 3). The maximum fresh weight (980 mg) occurred 20 days after the exponential growth phase. Then started to decline after 20 days. This was most likely due to the depletion of nutrients in the liquid MS medium, leading to cell death. This indicates that the optimum subculture interval for cell suspension cultures of gazania is 20 days, at this point the cells reach the progressive deceleration stage. In the present study, a sigmoid pattern of growth curve was observed in gazania with a maximum growth curve at 20 days of subculture and a minimum growth curve at 5 days of subculture.

### Effect of YE or MeJA elicitation on cell growth and custonolide accumulation (20-day culture)

The fresh biomass (730.7 and 748.0 g) was significantly higher in cell suspension cultures elicited with YE at a concentration of 200 mg/L and in the control-non-elicited treatment, respectively, showing non-significant difference. However, the growth of the dry biomass was significantly reduced after elicitation with YE, causing a 1.5–2-fold decrease (39.0–49.0 mg) as compared to the control (78.0 mg). Costunolide accumulation of 2.56–3.47 ppm in YE (100–250 mg/L) elicited cell suspension cultures were considerably higher than non-elicited ones (1.70 ppm). Specifically, 200 mg/L YE exhibited the highest costunolide accumulation differing significantly from the other treatments. There was a noticeable decline in cell growth of fresh biomass and costunolide content with 250 mg/L YE (one-way ANOVA) (Table 2).

**Table 2:** Effect of YE or MeJA concentration on cell growth parameters and costunolide accumulation

Treatments	Fresh weight (mg)	Dry weight (mg)	Costunolide (ppm)
YE (mg/L)			
0	748.0 ± 1.5^a(A)^	78.0 ± 0.6^a(A)^	1.70 ± 0.15^d(E)^
100	536.0 ± 5.0^c(D)^	43.0 ± 1.5^bc(DE)^	1.73 ± 0.01^d(E)^
150	364.0 ± 3.1^d(F)^	39.0 ± 1.7^c(EF)^	2.56 ± 0.01^c(D)^
200	730.7 ± 5.2^a(B)^	47.0 ± 3.1^b(CD)^	3.47 ± 0.01^a(B)^
250	670.7 ± 10.3^b(C)^	49.0 ± 3.5^b(C)^	3.02 ± 0.01^b(C)^
*p* values (one-way ANOVA) [YE concentration]	0.000***	0.000***	0.000***
MeJA (mg/L)			
0	748.0 ± 0.6^a(A)^	78.0 ± 0.6^a(A)^	1.70 ± 0.2^d(E)^
100	420.0 ± 1.5^b(E)^	58.0 ± 0.6^b(B)^	3.30 ± 0.6^b(B)^
150	301.7 ± 0.9^c(G)^	36.0 ± 0.0^c(F)^	5.50 ± 0.6^a(A)^
200	220.0 ± 3.6^d(H)^	28.0 ± 0.6^d(G)^	2.60 ± 0.2^c(D)^
250	130.0 ± 0.0^e(I)^	4.2 ± 0.2^e(H)^	1.60 ± 0.6^d(E)^
*p* values (one-way ANOVA) [MeJA concentration]	0.000***	0.000***	0.000***
Two-way ANOVA/general linear model			
Elicitor type (A)	0.000***	0.000***	0.000***
Elicitor concentration (B)	0.000***	0.000***	0.000***
(A)*(B)	0.000***	0.000***	0.000***

Means (*n* = 30) ± standard error (S.E.) with the same letter in a column are not statistically significant different from each other according to the Duncan’s multiple range test at *p* ≤ 0.05. ***p ≤ 0.001. Superscript small letters—differences between five concentrations for each elicitor type (either YE or MeJA) separately (one-way ANOVA). Superscript capital letters in parenthesis—differences between samples from the combined effect of two elicitor types and five elicitor concentrations (two-way ANOVA)

A significant and gradual decrease in the fresh and dry biomass of *G. rigens* cell suspension cultures was achieved in medium supplemented with increasing concentrations of MeJA (100, 150, 200, 250 mg/L) as compared to the control. Cell growth parameters (748.0 g fresh mass and 78.0 g dry mass) were the highest in the control treatment, while MeJA elicitation had a negative effect (decrease by 1.8–5.8 and 1.3–18.6 times in fresh and dry mass, respectively). Even though, MeJA elicitation adversely influenced cell growth, costunolide accumulation was enhanced by 100–200 mg/L MeJA. The largest increase in costunolide accumulation (5.50 ppm) was achieved on day 20 in medium fortified with 150 mg/L MeJA, being 3.2 times higher than in the non-elicited cells (1.70 ppm) (one-way ANOVA) (Table 2).

According to two-way ANOVA and General Linear Model, the effect of the main factors (elicitor type, elicitor concentration) and their interaction on cell growth parameters (fresh and dry biomass) and costunolide accumulation was significant (*p* = 0.000 < 0.05). Between the two elicitors and irrespective of their concentration, YE exhibited significantly higher fresh biomass yields in all concentrations tested than MeJA; however, both elicitors significantly suppressed fresh biomass. In the case of dry biomass, under the lowest concentration of 100 mg/L MeJA gave higher dry weight than YE, similar dry weight values were obtained by YE and MeJA when applied at 150 mg/L, whereas YE enhanced better dry biomass accumulation compared to MeJA at higher concentrations (200 and 250 mg/L) (Table 2).

### Effect of elicitation time combined with either 200 mg/L YE or 150 mg/L MeJA on cell growth and costunolide accumulation

The maximum costunolide accumulation (7.37 ppm) and fresh biomass yield (932.0 mg) of *G. rigens* from cell suspension cultures achieved after 15 and 20 days, respectively, of elicitation with 200 mg/L YE, being 2.12 and 1.28 times higher than the control on day 0 (3.47 ppm costunolide and 730.7 mg fresh mass), accordingly. Cell growth depicted by the dry biomass appeared to peak at day 5, whereas there was not a significant further increase in dry weights between the 5th and 20th day of elicitation (68.3–74.0 mg) (one-way ANOVA) (Table 3).

**Table 3:** Effect of elicitation time combined with 200 mg/L YE or 150 mg/L MeJA on cell growth parameters and costunolide accumulation

Treatments	Fresh weight (mg)	Dry weight (mg)	Costunolide (ppm)
YE elicitation time (days)			
0	730.7 ± 5.2^d(E)^	47.0 ± 3.1^b(D)^	3.47 ± 0.01^e(J)^
5	796.0 ± 2.5^c(D)^	68.3 ± 1.3^a(BC)^	4.98 ± 0.01^d(I)^
10	823.3 ± 3.5^b(C)^	70.0 ± 2.5^a(ABC)^	5.94 ± 0.01^b(F)^
15	784.7 ± 9.8^c(D)^	72.7 ± 1.3^a(ABC)^	7.37 ± 0.01^a(D)^
20	932.0 ± 0.6^a(B)^	74.0 ± 2.3^a(AB)^	5.60 ± 0.00^c(G)^
*p* values (one-way ANOVA) [YE, Elicitation time]	0.000***	0.000***	0.000***
MeJA elicitation time (days)			
0	302.0 ± 0.6^d(G)^	36.0 ± 0.0^c(E)^	5.50 ± 0.06^e(H)^
5	543.0 ± 1.5^c(F)^	66.0 ± 2.1^b(C)^	13.94 ± 0.01^b(B)^
10	732.0 ± 1.2^b(E)^	72.7 ± 2.9^ab(ABC)^	18.47 ± 0.02^a(A)^
15	954.0 ± 2.3^a(A)^	76.3 ± 3.2^a(A)^	10.29 ± 0.01^c(C)^
20	951.0 ± 2.6^a(A)^	73.0 ± 1.2^ab(ABC)^	7.11 ± 0.01^b(E)^
*p* values (one-way ANOVA) [MeJA, Elicitation time]	0.000***	0.000***	0.000***
Two-way ANOVA/general linear model			
Elicitor type (A)	0.000***	*0.266 ns*	0.000***
Elicitation time (B)	0.000***	0.000***	0.000***
(A)*(B)	0.000***	0.026*	0.000***

Means (*n* = 30) ± standard error (S.E.) with the same letter in a column are not statistically significant different from each other according to the Duncan’s multiple range test at *p* ≤ 0.05. ns *p* > 0.05; *p ≤ 0.05; ***p ≤ 0.001. Superscript small letters—differences between five elicitation times for each elicitor type (either YE or MeJA) separately (one-way ANOVA). Superscript capital letters in parenthesis—differences between samples from the combined effect of two elicitor types and five elicitation times (two-way ANOVA)

Considering the weight of cells after each 5-day interval in medium supplemented with 150 mg/L MeJA, we observed that the cells grew well for 20 days and their fresh weight increased significantly with incubation time. Fresh mass of cells was maximum (951.0 mg) after elicitation with MeJA and 20 days of culture, being 3.15 times higher compared with elicited-cells on day 0 (302.0 mg). Among the different elicitation exposure periods, the day 15 was the optimum time for dry biomass accumulation (76.3 mg), being 2.1-fold higher than on day 0. The treatment of MeJA (150 mg/L) to *G. rigens* suspension cell cultures led to a significant increase in custonolide accumulation over the experimental period (5–20 days) relative to control. In particular, the largest custonolide content was obtained with 150 mg/L MeJA for 10 days (18.47 ppm), being 3.36 times higher compared to day 0 (one-way ANOVA) (Table 3).

According to two-way ANOVA and General Linear Model, the effect of the main factors (elicitor type, elicitation time) and their interaction on fresh biomass and costunolide accumulation was significant (*p* = 0.000 < 0.05). In the case of dry biomass, the elicitation time as a single factor and its interaction with elicitor type showed significant effect (*p* = 0.000 and 0.026 < 0.05) in contrast to the non-significant effect of the elicitor type as a single factor (*p* = 0.266 > 0.05). The comparison between the two elicitors and irrespective of elicitation culture period clearly showed that cells elicited with YE for 0–10 days gave higher fresh weights than did with MeJA; however, MeJA drastically increased fresh weight much higher as compared to YE at longer elicitation periods (10–20 days). Under the same elicitation time, no substantial differentiations were observed in dry weights between the two elicitors (YE, MeJA) tested; however, dry weight was maximized after elicitation with either YE or MeJA for 10–20 days. MeJA exhibited significantly higher costunolide content than YE for all elicitation periods, while its accumulation was maximized after treatment of cells with 150 mg/L MeJA for 10 days (Table 3).

## Discussion

### Callus induction

In the present study with *G. rigens*, the type of explant and 2,4-D concentration had an effect on the type and morphology of callus formation. Alterations in the color of callus in *G. rigens* under study can be attributed to interactions performed among the level of endogenous and exogenous plant growth regulators, different explant types (leaf, stem) and micro-environment during culture including temperature, photoperiod duration and light intensity (Ellias et al. 2014). The different reaction of explant types to callus induction would be ascribed to the balance of endogenous hormones inside plant tissues, since segments taken from the leaf base have more juvenile and lower number of differentiated cells, exhibiting higher meristematic activity and callusing potential (Asghari et al. 2012) as well as higher number of receptors for the growth regulator present in the culture medium (Close and Gallagher-Ludeman 1989), as compared to the leaf apex and middle segments. Auxins, especially 2,4-D play an important role in callus induction (Baskaran et al. 2006), especially of soft/friable callus by increasing the size of vacuoles (Borejsza-Wysoki and Hrazdin 1994). The increase of 2,4-D concentration in the medium was accompanied by an analogous increase in callusing response (Hassan et al. 2009); however, higher 2,4-D concentrations have been reported to be associated with reduced callogenic responses (%) in different explant types depended on plant species (Ali and Afrasiab 2014; Dangash et al. 2015). Stem explants of the studied *G. rigens* treated with 1.5 mg/L 2,4-D followed by leaf explants with 1 mg/L 2,4-D were the most effective treatments for callus induction (90% and 80%, respectively). Similarly, high percentages of friable callus induction (82.5–100%) after culture of stem explants in MS medium containing 2,4-D (0.5–0.75 mg/L) have also been reported in other Asteraceae species, including *Artemisia annua* L. (Dangash et al. 2015) and *Achyrocline flaccida* (Weinm.) DC. (Bonnecarrère et al. 2009). On the contrary, leaves served as better explants than stems for callus induction under the effect of 2,4-D either alone in other plant species including *Ocinum tenuiflorum* (Sharan et al. 2018) and safflower (*Carthamus tinctorius*) (Ali and Afrasiab 2014) or combined with thidiazuron (TDZ) in feverfew (*Tanacetum parthenium*) (Mahood et al. 2022). In the studied *G. rigens*, callus quality (color, texture, growth rate) was superior when calli derived from stem explants (friable, white–yellow, fast-growing). It has been shown that the friability of callus positively affects the successful response of cell suspension cultures based on the fact that the cream colored friable callus undergo successive sub-cultures results in the formation of fine cells appropriate for suspension culture (Bhojwani and Razdan 1996).

### Cell suspension cultures growth curve

In the present study, *G. rigens* cell suspension cultures were established from friable callus derived from stem explants treated with 1.5 mg/L 2,4-D based on the fact that higher cell division rates can be obtained in cell suspension cultures as compared to cell callus cultures (Mustafa et al. 2011). The knowledge on cell suspension growth curve has been reported to be an essential step for logarithmic growth maintenance (Bona et al. 2012) with 2,4-D to be the most common used auxin for the establishment of cell suspension cultures (Szabados et al. 1991). Our results are partly in agreement (explant type, 2,4-D concentration) with those reported in *Achyrocline flaccida*, where cell suspension cultures established from friable callus originated from leaf explants on MS medium containing 0.5 mg/L 2,4-D (Bonnecarrère et al. 2009).

In this study, a sigmoid pattern of growth curve was observed in gazania with a maximum growth curve at 20 days of subculture and a minimum growth curve at 5 days of subculture, as shown by fresh weight values, indicating that 20 days is the critical point the cells reach the progressive deceleration stage. Similar growth curve sigmoid pattern to the *G. rigens* under study has been reported for *Ocinum tenuiflorum* cells (lag phase: 0–5 days, logarithmic phase: 5–20th day, stationary phase: 20–25th day) (Sharan et al. 2021). In *Achyrocline flaccida*, the exponential phase of cell suspension cultures in DKW medium enriched with 2,4-D was longer (10 days) and the fresh weight at the end of the period higher (Bonnecarrère et al. 2009). Possible explanations for the decline in cell biomass and deceleration in growth of cell suspension cultures of *G. rigens* after the 20th day can be the growth reduction because of cell signaling and accumulation of toxic substances, cell death linked to oxygen depletion, limited air availability and gas exchange, nutrients consumption, smaller physical area (Bona et al. 2012) as well as the changes in the pH value of the spent medium during different growth phases of suspension cell culture, which is species-depended (Santos et al. 2010).

The maximum fresh weight of gazania cells suspension cultures occurred 20 days after the exponential growth phase and then started to decline. In three different species of the genus *Ocinum*, the maximum fresh weight accumulation was observed on day 14 for *O. basilicum*, on day 32 for *O. sanctum* and on day 28 for *O. gratissimum* (Mathew and Sankar 2012). The fresh weight of the cell suspension culture in two *Calendula* species (*C. officinalis* and *C. arvensis*, also Asteraceae) reached the maximum and at the same time constant value between the 25th and the 30th day, wherein a significant decrease in biomass accumulation was recorded after the 30th day and until the end of the 40th-day culture period (lag: 0–5 days, log: 5–25 days, and death phase: 25–30 days) (Kaya 2019). Different growth curve than that presented in this study with gazania was recorded in the case of *Iphiona mucronate,* where the peak in fresh weight was noticed on day 9 (lag: 2 days, exponential phase: 2–6 days, stationary: 6–9 days and death phase: after 15 days with browning of suspension cultures) (Al-Gendy et al. 2015). During the progress of growth phases, the decline in pH of the medium could be the outcome of the uptake of ammonium (NH_4_^+^) and the liberation of H^+^ ions, whereas the increase in pH is ascribed to the rather higher assimilation of nitrate (NO_3_^−^) by the cultures than the NH_4_^+^ (Santos et al. 2010), leading cell suspension cultures to growth inhibition, browning and finally to cell death.

### Effect of YE elicitation on cell growth and custonolide accumulation (20-day culture)

The accumulation of valuable SMs with commercial application potential to the bio-industry can be enhanced via the use of in vitro stress factors called elicitors (Murphy et al. 2007), as signaling molecules triggering the formation of bioactive compounds (Açıkgöz et al. 2019), activating the plant’s defense response (Jiao et al. 2016) and initiating the complex signal transduction network involving regulation of gene expression responsible for biosynthesis of targeted SMs (Savitha et al. 2006). There are several factors influencing the efficacy of elicitation in plants including the elicitor’s specificity, concentration, and exposure time, culture conditions (nutrient composition of the medium, growth regulators, light) and cell culture growth stages (Wiktorowska et al. 2010). The cell biomass is an essential factor to measure growth rate, and the concentration of the selected elicitor species-depended of paramount significance, since concentrations higher than the optimum can lead to hypersensitive response and cell death (Park et al. 2020). Elicitors such as YE stimulate the production of specific and targeted SMs, presumably by mimicking a pathogenic fungal infection (Li and Barz 2006).

In the present study with *G. rigens*, the fresh biomass and costunolide accumulation was higher in cell suspension cultures elicited with 200 mg/L YE and in the non-elicited cells. The stimulating effect of yeast extract elicitation on cell growth and biomass production has been reported in several plant species including *Gentiana dinarica* (Krstić-Milošević et al. 2017), *Stevia rebaudiana* (Bayraktar et al. 2016), *Salvia castanea* (Li et al. 2016), *Ophiorrhiza mungos* (Deepthi and Satheeshkumar 2016), and *Panax vietnaminis* (Trong et al. 2017). However, the application of YE (50–200 mg/L) in gazania had an inhibitory effect on dry biomass after a 20-day period. Contradictory results to ours in gazania have been reported in date palm, since cell suspension cultures elicited with YE (50–150 mg/L) performed increased dry biomass yield with the increase in the elicitor’s concentration (Al-Khayri and Naik 2020).

It is clearly supporting the concentration-dependent effect of elicitors on biomass and bioactive compounds production (Ho et al. 2018). Costunolide accumulation in YE (100–250 mg/L) elicited cell suspension cultures of gazania were considerably higher than non-elicited ones. In *Ocimum tenuiflorum* L., YE at 50 mg/L was optimal for inducing significantly higher accumulation of the targeted bioactive compound (Sharan et al. 2021). Taking into consideration the simultaneous maximum increase in cell growth/fresh biomass and costunolide accumulation of cell suspension cultures of gazania under study, 200 mg/L YE was the optimum concentration after a 20-day period. YE is the water-soluble portion of autolyzed yeast and it can provide essential vitamins, nitrogen, amino acids, peptides, carbohydrates, and some growth regulators (Mosser et al. 2011), functioning as a bio-enhancer of plant growth or the biosynthesis of plant pigments and other bioactive compounds (Złotek 2017) related to FPS gene expression and mediated by reactive oxygen species signaling and jasmonic acid signal transduction (Rahimi et al. 2015). The stimulating effect of YE on biomass and SMs in in vitro cultures can also be explained by the presence of some cations such as Ca^2+^, Co^2+^ and Zn^2+^ in YE exerting abiotic elicitors action (Sharan et al. 2018) and the complex YE-induced stress response in the cultures, such as lipid peroxidation and metabolic pathways activation (Sánchez-Sampedro et al. 2005).

### Effect of MeJA elicitation on cell growth and custonolide accumulation (20-day culture)

In the current study employing cell suspension cultures of gazania, elicitation with MeJA (100–250 mg/L) negatively affected cell growth parameters (fresh and dry biomass) after a 20-day period. In line with our findings, the increase in MeJA concentration showed a clear repression of cell growth and gradual decrease in accumulated biomass for three species of the genus *Ocinum* (*O. basilicum*, *O. sanctum* and *O. gratissimum*) (Mathew and Sankar 2012), in *Panax ginseng* (Ali et al. 2007), *Mentha piperita* (Krzyzanowska et al. 2012) and *Ginkgo biloba* (Kang et al. 2006), which was proportional to the applied concentration of MeJA. In all these studies, the decline in biomass accumulation may be due to toxicity stress induced by high concentrations and prolonged exposure of MeJA (Veerashree et al. 2012), leading to cell death (Rijhwani and Shanks, 1998). Our results corroborate the studies conducted in *Changium smyrnioides* (Cai et al. 2017) and *Mentha piperita* (Krzyzanowska et al. 2012), where suspension cells elicited with MeJA and jasmonic acid (JA), respectively, showed inhibition of biomass accumulation and suppressed cell growth. In contrast to our results in gazania, MeJA elicitation has been illustrated to have a stimulating effect on cell growth and biomass production in other Asteraceae species including *Lavandula vera* MM (Georgiev et al. 2007), *Achillea gypsicola* (Açıkgöz et al. 2019), and *Helichrysum stoechas* (Gourguillon et al. 2022).

According to Jong-Joo and Yang (2003), MeJA is competent of upregulating genes participated in jasmonate biosynthesis, secondary metabolism, cell wall biosynthesis and tolerance to biotic and/or abiotic stress conditions. Even though, MeJA elicitation adversely influenced cell growth of gazania suspension cultures, costunolide accumulation was enhanced by 100–200 mg/L MeJA, being maximum at 150 mg/L. Based on the observations of Suzuki et al. (2005), there is an inverse relationship between biomass and secondary metabolites production, as MeJA application resulted in cell growth inhibition due to toxicity and excited the accumulation of bioactive compounds due to inability to activate the genes involved in the phenylpropanoid/flavonoid pathway. Similarly, explants after elicitation with MeJA showed considerably higher scavenging free radical activity as compared to the non-elicited ones (Shilpha et al. 2015). Consistent with our results in gazania regarding costunolide accumulation, the positive effect of MeJA elicitation on targeted secondary metabolites in other Asteraceae species has been reported including 3,5-diCQA (main phenolic acid) in *Helichrysum stoechas* cells (Gourguillon et al. 2022), and artemisinin, artemisinic acid, dihydroartemisinic acid, and other sesquiterpenoids as well as triterpenoids in *Artemisia annua* L. (Wang et al. 2009).

### Effect of elicitation time combined with either 200 mg/L YE or 150 mg/L MeJA on cell growth and costunolide accumulation

There are various factors that enhance the effective role of elicitors on cell growth, culture viability, biomass and secondary metabolites production, such as the age of the cell culture, elicitation time, elicitor type (biotic or abiotic), elicitor concentration and growth stage of the cultures (Açıkgöz 2020). In this study, costunolide accumulation and cell growth (fresh biomass) were substantially enhanced after 15 and 20 days of elicitation with 200 mg/L YE, respectively. The stronger stimulation of secondary metabolites by fungal elicitor (e.g., YE) often occurs in the late exponential growth stage and early stationary phase (Kitamura et al. 1998). In accordance with our findings, the highest increase in biomass production of *Ocinum tenuiflorum* was noted when suspension cultures exposed to 50 mg/L YE for 4 days (Sharan et al. 2021). Dry biomass of gazania cells elicited with YE reached their maximum competence after 5 days of culture, which remained constant until the end of the experimental period (20 days). Similarly, in other Asteraceae species, YE elicitation of cell suspension cultures in different exposure times has been linked to increased biomass and secondary metabolites production including *Silybum marianum* (L.) Gaertn (Asteraceae) showing maximum cell dry weight with 80 mg/L YE for 24 h and highest silymarin content (flavonolignans) with 120 mg/L YE for 72 h (Rahimi Ashtiani et al. 2009) and *Iphiona mucronata* showing highest flavonoids and phenolics production with 10 mg/l YE for 12 h (Al-Gendy et al. 2015).

Among the 5 different elicitation exposure periods to MeJA, day 10, day 15 and day 20 gave the highest costunolide content, dry biomass and fresh biomass production of gazania cell suspension cultures, accordingly. Therefore, it is clearly illustrated that the biosynthesis of many secondary metabolites such as sesquiterpenes (e.g., costunolide) is triggered by MeJA application to the culture medium (Matkowski 2008). In feverfew (*Tanacetum parthenium*) hairy root cultures, all applied elicitors (2.5 mg/L YE, 20–25 mg/L MeJA, YE + MeJA) increased parthenolide (PN) (a sesquiterpene lactone compound) production, being maximum after treatment with YE + MeJA for 48 h (Pourianezhad et al. 2019a,b). Among the wide range of elicitors, YE and MeJA have been extensively used due to their ability to induce the biosynthesis of plant pro-health compounds, such as vitamins, plant pigments, essential oils, or phenolic compounds (Złotek et al. 2016). In several other Asteraceae species, MeJA concentration, elicitation time as well as their interaction play a key role exerting different responses related to cell growth parameters (biomass yields) and targeted bioactive compounds which are species-specific- and genotype-depended. In particular, MeJA was found to enhance accumulated cell biomass in three different *Ocinum* species, including *O. basilicum* (25 μM MeJA, 12 h), *O. sanctum* (25 μM MeJA, 48 h) and *O. gratissimum* (50 μM MeJA, 8 h) (Mathew and Sankar 2012). Concerning secondary metabolites accumulation in cell suspension cultures, increased rhamnetin production in *Vernonia anthelmintica* (L.) Willd. was obtained after treatment with 180 mg/L MeJA for 6 days (Rajan et al. 2020), while flavonoids and phenolics level in *Iphiona mucronata* (Forssk.) Asch. & Schweinf was highest with 150 μM MeJA for 6 h (Al-Gendy et al. 2015).

## Conclusions

It this study with *G. rigens*, the type of explant, and the concentration of 2,4-D had an effect on the type, morphology and callus induction percentage. Stem explants proved to be the best source for further establishment of cell suspension cultures. Cell suspension culture appears to be a promising technique for in vitro accumulation of valuable secondary metabolites and elicitation a proven strategy for improving their production yields. The results recommend the use of elicitation (MeJA or YE) as an effective method to raise costunolide content and biomass accumulation in cell suspension cultures of *G. rigens* based on the better understanding of how elicitors affect bioactive compounds in an attempt to select elicitors for enhancing production of sesquiterpene lactones, e.g., costunolide at industrial scale. Biomass cell growth and costunolide production was seen to be depended on elicitor type, elicitor concentration, and elicitation time as well as on their interaction effects. Particularly, MeJA was more effective elicitor type than YE after 20 days of culture, regardless of concentrations applied. In particular, the optimum concentration for each elicitor type was different; 150 mg/L for MeJA and 200 mg/L for YE. Taking into consideration the elicitation time as well, cell growth was better enhanced after 15 days of culture in liquid medium enriched with 150 mg/L for MeJA, while elicited cells for 10 days exhibited the highest costunolide accumulation. To the best of our knowledge, there is no report for elicitation in the genus *Gazania*; therefore, the original aspect of the present work is that for the first time an elicitor-enhanced metabolites production in *G. rigens* is reported. The results of the present study demonstrated that by optimizing the concentrations of the elicitors and the exposure time of elicitation, it is possible to produce the desired secondary metabolites of *G. rigens* in in vitro laboratory conditions. Further research is needed to optimize the best and reproducible protocols for scale-up culture in bioreactors for increased accumulation of secondary metabolites. In this context, understanding the metabolic pathways leading to the production of targeted bioactive compounds and their regulation is indispensable. The acquisition of further referential information on the enzymes and genes involved as well as the transcription factors controlling these pathways could be an extra advantage in the development of more efficient elicitation strategies of *G. rigens* secondary metabolism.

## Data Availability

This manuscript has no associated data. All data generated or analyzed during this study are included in this published article.

## References

[CR1] Abd El-Salam M, Mekky H, El-Naggar EMB, Ghareeb D, El-Demellawy M, El-Fiky F (2015). Hepatoprotective properties and biotransformation of berberine and berberrubine by cell suspension cultures of *Dodonaea viscosa* and *Ocimum basilicum*. S Afr J Bot.

[CR2] Açıkgöz MA (2020). Establishment of cell suspension cultures of *Ocimum basilicum* L. and enhanced production of pharmaceutical active ingredients. Ind Crop Prod.

[CR3] Açikgöz MA, Kara SM, Aygün A, Özcan MM, Bati AYE (2019). Effects of methyl jasmonate and salicylic acid on the production of camphor and phenolic compounds in cell suspension culture of endemic Turkish yarrow (*Achillea gypsicola*) species. Turk J Agric For.

[CR4] Al-Gendy AA, Bakr RO, El-Gindi OD (2015). Production of flavonoids and phenolic compounds by elicitation of Iphiona mucronata (Forssk.) Asch. &amp; Schweinf (Asteraceae) callus and suspension cultures. Int J Pharmacognosy Phytochem.

[CR5] Ali N, Afrasiab H (2014). Effect of TIBA and other plant growth regulators on callogenic response from different explants of safflower (*Carthamus tinctorius*). Int J Agric Biol.

[CR6] Al-Khayri JM, Naik PM (2020). Elicitor-induced production of biomass and pharmaceutical phenolic compounds in cell suspension culture of date palm (*Phoenix dactylifera* L.). Molecules.

[CR7] Asghari F, Hossieni B, Hassani A, Shirzad H (2012). Effect of explants source and different hormonal combinations on direct regeneration of basil plants (*Ocimum basilicum* L.). Aust J Agr Eng.

[CR8] Baenas N, Garcia-Viguera C, Moreno D (2014). Elicitation: a tool for enriching the bioactive composition of foods. Molecules.

[CR9] Baskaran P, Raja Rajeswari B, Jayabalan N (2006). Development of an in vitro regeneration system in sorghum [*Sorghum bicolor* (L.) Moench] using root transverse thin cell layers (tTCLs). Turk J Bot.

[CR10] Bayraktar M, Naziri E, Akgun IH, Karabey F, Ilhan E, Akyol B, Bedir E, Gurel A (2016). Elicitor induced stevioside production, in vitro shoot growth, and biomass accumulation in micropropagated *Stevia rebaudiana*. Plant Cell Tiss Organ Cult.

[CR11] Bhojwani SS, Razdan MK (1996). Plant tissue culture: theory and practices, a revised edition. Studies in plant science.

[CR12] Boller T (1995). Chemoperception of microbial signals in plant cells. Annu Rev Plant Physiol Plant Mol Biol.

[CR13] Bona CM, Santos GD, Biasi LA (2012). *Lavandula* calli induction, growth curve and cell suspension formation. Rev Bras Cienc Agrar.

[CR14] Bonnecarrère V, Berná L, Castillo A (2009). Establishment of micropropagation and cell suspension culture conditions on *Achyrocline flaccida* (Weinm.) DC. (Asteraceae). Agrociencia Uruguay.

[CR15] Borejsza-Eysocki W, Hrazdin G (1994). Establishment of callus and cell suspension cultures of raspberry (*Rubus idaues* cv. Royalty). Plant Cell Tiss Organ Cult.

[CR16] Cai Z, Kastell A, Mewis I, Knorr D, Smetanska I (2012). Polysaccharide elicitors enhance anthocyanin and phenolic acid accumulation in cell suspension cultures of *Vitis vinifera*. Plant Cell Tiss Organ Cult.

[CR17] Cai J, Ma Y, Hu P, Zhang Y, Chen J, Li X (2017). Elicitation of furanocoumarins in *Changium smyrnioides* suspension cells. Plant Cell Tiss Organ Cult.

[CR18] Chan LK, Lim PS, Choo ML, Boey PL (2010). Establishment of *Cyperus aromaticus* cell suspension cultures for the production of Juvenile hormone III. In Vitro Cell Dev Biol Plant.

[CR19] Close KR, Gallagher-Ludeman LA (1989). Structure-activity relationships of auxin-like plant growth regulators and genetic influences on the culture induction responses in maize (*Zea mays* L.). Plant Sci.

[CR20] Damayanti F, Indrianto A, Sasongko AB, Fajarina S, Prabowo BH, Iskandar A, Hidayati L, Tunjung WAS (2020) Variation of 2,4-dichlorophenoxyacetic acid (2,4-D) concentration on kaffir lime callus growth as raw material for cell suspension. The 6th International Conference on Biological Science ICBS 2019, AIP Conference Proceedings 2260:030012. 10.1063/5.0016420

[CR21] Dangash A, Ram M, Niranjan R, Bharillya A, Misra H, Neeta Pandya N, Jain DC (2015). In vitro selection and hormonal regulation in cell culture of *Artemisia annua* L. Plant JSM Cell Dev Biol.

[CR22] de Kraker JW, Franssen MCR, Joerink M, de Groot A, Bouwmeester HJ (2002). Biosynthesis of costunolide, dihydrocostunolide, and leucodin. Demonstration of cytochrome P450-catalyzed formation of the lactone ring present in sesquiterpene lactones of chicory. Plant Physiol.

[CR23] Deepthi S, Satheeshkumar K (2016). Enhanced camptothecin production induced by elicitors in the cell suspension cultures of *Ophiorrhiza mungos* Linn. Plant Cell Tiss Organ Cult.

[CR24] Elias H, Taha RM, Hasbullah NA, Mohamed N, Manan AA, Mohamed N, Mohajer S (2014). The effects of plant growth regulators on shoot formation regeneration and colored callus production in *Echinocereus cinerascens *in vitro. Plant Cell Tiss Organ Cult.

[CR25] Farjaminezhad R, Garoosi G (2021). Improvement and prediction of secondary metabolites production under yeast extract elicitation of *Azadirachta indica* cell suspension culture using response surface methodology. AMB Expr.

[CR26] George EF, Hall MA, De Klerk G (2008). Plant propagation by tissue culture.

[CR27] Georgiev MI, Kuzeva SL, Pavlov AI, Kovacheva EG, Illieva MP (2007). Elicitation of rosmarinic acid by *Lavendula vera* MM cell suspension culture with abiotic elicitors. World J Microb Biotechnol.

[CR28] Gourguillon L, Rustenholz C, Le Gélébart E, Lobstein A, Gondet L (2022). Methyl jasmonate elicited *Helichrysum stoechas* (L.) Moench cell suspensions, a promising source of extracts with allelopathic activity?. JOJ Hortic Arboric.

[CR29] Hassan MM, Azam FMS, Chowdhury MH, Rahmatullah M (2009). Callus induction of *Abrus precatorius*: screening of phytohormones. Am-Eurasian J Sustain Agric.

[CR30] Ho TT, Lee JD, Jeong CS, Paek KY, Park SY (2018). Improvement of biosynthesis and accumulation of bioactive compounds by elicitation in adventitious root cultures of *Polygonum multiflorum*. Appl Microbiol Biotechnol.

[CR31] Jiao J, Gai QY, Wang W, Luo M, Zu YG, Fu YJ, Ma W (2016). Enhanced astragaloside production and transcriptional responses of biosynthetic genes in *Astragalus membranaceus* hairy root cultures by elicitation with methyl jasmonate. Biochem Eng J.

[CR32] Jong-Joo C, Yang DC (2003). Methyl jasmonate as a vital substance in plants. Trends Genet.

[CR33] Kang SM, Min JY, Kim YD, Kang YM, Park DJ, Jung HN, Kim SW, Choi MS (2006). Effects of methyl jasmonate and salicylic acid on the production of biloalide and ginkgolides in cell cultures of *Ginkgo biloba*. In Vitro Cell Dev Biol-Plant.

[CR34] Kaya N (2019). The effect of some plant growth regulators on cell biomass in the cell suspension culture of *Calendula officinalis* L. and *Calendula arvensis* L. species. Int J Sci Res.

[CR35] Kieran PM, MacLoughlin PF, Malone DM (1997). Plant cell suspension cultures: some engineering considerations. J Biotechnol.

[CR36] Kim DY, Choi BY (2019). Costunolide—a bioactive sesquiterpene lactone with diverse therapeutic potential. Int J Mol Sci.

[CR37] Kitamura Y, Ikenaga T, Ooe Y, Hiraoka N, Mizukami H (1998). Induction of furanocoumarin biosynthesis in *Glehnia littoralis* cell suspension cultures by elicitor treatment. Phytochemistry.

[CR38] Krstić-Milošević D, Janković T, Uzelac B, Vinterhalter D, Vinterhalter B (2017). Effect of elicitors on xanthone accumulation and biomass production in hairy root cultures of *Gentiana dinarica*. Plant Cell Tiss Organ Cult.

[CR39] Krzyzanowska J, Czubacka A, Pecio L, Przybys M, Doroszewska T, Stochmal A, Oleszek W (2012). The effects of jasmonic acid and methyl jasmonate on rosmarinic acid production in *Mentha piperita* cell suspension cultures. Plant Cell Tiss Organ Cult.

[CR40] Li YF (2011) Study on introduction and application of ground-cover plant *Gazania rigens* L. in Suzhou area. Dissertation, Soochow University, Suzhou (**in Chinese**)

[CR41] Li WW, Barz W (2006). Structure and accumulation of phenolics in elicited *Echinacea purpurea* cell cultures. Planta Med.

[CR42] Li B, Wang B, Li H, Peng L, Ru M, Liang Z, Yan X, Zhu Y (2016). Establishment of *Salvia castanea* Diels F. *tomentosa* Stib. hairy root cultures and the promotion of tanshinone accumulation and gene expression with Ag+, methyl jasmonate, and yeast extract elicitation. Protoplasma.

[CR43] Mahadi I, Syafi’i W, Sari Y (2016). Callus induction of calamansi (*Citrus microcarpa*) using 2,4-D and BAP hormones by in vitro methods. Indones J Agric Sci.

[CR44] Mahood HE, Abbas MK, Zahid NA (2022). Micropropagation of feverfew (*Tanacetum parthenium*) and quantification of parthenolide content in its micropropagated and conventionally grown plants. Horticulturae.

[CR45] Majdi M, Liu Q, Karimzadeh G, Malboobi MA, Beekwilder J, Cankar K, Vos Rd, Todorović S, Simonović A, Bouwmeester H (2011). Biosynthesis and localization of parthenolide in glandular trichomes of feverfew (*Tanacetum parthenium* L. Schulz Bip.). Phytochemistry.

[CR46] Mathew R, Sankar DP (2012). Effect of methyl jasmonate and chitosan on growth characteristics of *Ocimum basilicum* L., *Ocimum sanctum* L. and *Ocimum gratissimum* L. cell suspension cultures. Afr J Biotechnol.

[CR47] Matkowski A (2008). Plant in vitro culture for the production of antioxidants—a review. Biotechnol Adv.

[CR48] Mosser M, Kapel R, Aymes A, Bonanno LM, Olmos E, Besançon I, Druaux D, Chevalot I, Marc I, Marc A (2011). Characterization of chromatographic yeast extract fractions promoting CHO cell growth. BMC Proc.

[CR49] Mulabagal V, Tsay HS (2004). Plant cell cultures—an alternative and efficient source for the production of biologically important secondary metabolites. Int J Appl Sci Eng.

[CR50] Murashige T, Skoog F (1962). A revised medium for rapid growth and bioassays with tobacco tissue cultures. Physiol Plant.

[CR51] Murphy T, Parra R, Radman R, Roy I, Antony HA, Dixon K, Keshavarz T (2007). Novel application of oligosaccharides as elicitors for the enhancement of bacitracin A production in cultures of *Bacillus licheniformis*. Enzyme Microb Technol.

[CR52] Murthy HN, Lee EJ, Paek KY (2014). Production of secondary metabolites from cell and organ cultures: strategies and approaches for biomass improvement and metabolite accumulation. Plant Cell Tiss Organ Cult.

[CR53] Muschietti LV, Ulloa JL (2016). Natural sesquiterpene lactones as potential trypanocidal therapeutic agents: a review. Nat Prod Commun.

[CR54] Mustafa NR, de Winter W, van Iren F, Verpoorte R (2011). Initiation, growth and cryopreservation of plant cell suspension cultures. Nat Protoc.

[CR55] Nagella P, Murthy HN (2011). Effects of macroelements and nitrogen source on biomass accumulation and withanolide: a production from cell suspension cultures of *Withania somnifera* (L.) Dunal. Plant Cell Tiss Organ Cult.

[CR56] Nasim SA, Dhir B, Kapoor R, Fatima S, Mujib A (2010). Alliin production in various tissues and organs of *Allium sativum* grown under normal and sulphur-supplemented *in vitro* conditions. Plant Cell Tiss Organ Cult.

[CR57] Park YJ, Kim JK, Park SU (2020). Yeast extract improved biosynthesis of astragalosides in hairy root cultures of *Astragalus membranaceus*. Prep Biochem Biotech.

[CR58] Pourianezhad F, Rahnama H, Mousavi A, Khosrowshahli M, Mafakheri S (2019). Effects of combined elicitors on parthenolide production and expression of parthenolide synthase (TpPTS) in *Tanacetum parthenium* hairy root culture. Plant Biotechnol Rep.

[CR59] Pourianezhad F, Rahnama H, Mousavi A, Khosrowshahli M, Mafakheri S (2019). Parthenolide production in cell suspension culture of feverfew. Bioresour Bioprocess.

[CR60] Rahimi Ashtiani S, Hasanloo T, Bihamta Mohammad R (2009). Using yeast extract as an approach to increase flavonolignans content in cell suspension culture of milk thistle plant via elicitation mechanism. J Med Plants.

[CR61] Rahimi S, Kim YJ, Yang DC (2015). Production of ginseng saponins: elicitation strategy and signal transductions. Appl Microbiol Biotechnol.

[CR62] Rajan M, Feba KS, Chandran V, Shahena S, Mathew L (2020). Enhancement of rhamnetin production in *Vernonia anthelmintica* (L.) Willd. cell suspension cultures by eliciting with methyl jasmonate and salicylic acid. Physiol Mol Biol Plants.

[CR63] Rani D, Meelaph T, De-Eknamkul W, Vimolmangkang S (2020). Yeast extract elicited isoflavonoid accumulation and biosynthetic gene expression in *Pueraria candollei* var. *mirifica* cell cultures. Plant Cell Tiss Organ Cult.

[CR64] Rao S, Ravishankar GA (2002). Plant cell cultures: chemical factories of secondary metabolites. Biotechnol Adv.

[CR65] Rao AS, Kelkar G, Bhattacharyya S (1960). Terpenoids-XXI: The structure of costunolide, a new sesquiterpene lactone from costus root oil. Tetrahedron.

[CR66] Rasul A, Parveen S, Ma T (2012). Costunolide: a novel anti-cancer sesquiterpene lactone. Bangladesh J Pharmacol.

[CR67] Rijhwani SK, Shanks JV (1998). Effect of elicitor dosage and exposure time on biosynthesis of indole alkaloids by *Catharanthus roseus* hairy root cultures. Biotechnol Progr.

[CR68] Sánchez-Sampedro MA, Fernández-Tárrago J, Corchete P (2005). Yeast extract and methyl jasmonate-induced silymarin production in cell cultures of *Silybum marianum* (L.) Gaertn. J Biotechnol.

[CR69] Santos ALWD, Silveira V, Steiner N, Maraschin M, Guerra MP (2010). Biochemical and morphological changes during the growth kinetics of *Araucaria angustifolia* suspension cultures. Braz Arch Biol Technol.

[CR70] Savitha BC, Thimmaraju R, Bhagyalakshmi N, Ravishnkar GA (2006). Different biotic and abiotic elicitors influence betalain production in hairy root cultures of *Beta vulgaris* in shake flask and bioreactor. Process Biochem.

[CR71] Sharan S, Mukhopadhyay K, Sarin NB (2018). Establishment of *in vitro* callus cultures and comparative phytochemical study of in vitro callus cultures and field grown plants of *Ocimum tenuiflorum* L. Plant Arch.

[CR72] Sharan S, Sharin NB, Mukhopadhyay K (2021). Development of an elicitation strategy on enhanced accumulation of oleanolic acid in suspension cultures of *Ocimum tenuiflorum* L. Res Sq.

[CR73] Shilpha J, Satish L, Kavikkuil M, Largia MJV, Ramesh M (2015). Methyl jasmonate elicits the solasodine production and anti-oxidant activity in hairy root cultures of *Solanum trilobatum* L. Ind Crops Prod.

[CR74] Stephane FFY, Jules BKJ, de Oliveira MS, Silva S, Da Costa WA (2020). Terpenoids as important bioactive constituents of essential oils. Essential oils: bioactive compounds, new perspectives and applications.

[CR75] Suzuki H, Reddy MS, Naoumkina M, Aziz N, May GD, Huhman DV, Sumner LW, Blount JW, Mendes P, Dixon RA (2005). Methyl jasmonate and yeast elicitor induce differential transcriptional and metabolic re-programming in cell suspension cultures of the model legume *Medicago truncatula*. Planta.

[CR76] Szabados L, Mroginski LA, Roca WM, Roca WM, Mroginski LA (1991). Suspensiones celulares: descripción, manipulación y aplicaciones. En cultivos de tejidos en la agricultura fundamentos y aplicaciones.

[CR77] Thiruvengadam M, Kim SH, Chung IM (2015). Exogenous phytohormones increase the accumulation of health-promoting metabolites, and influence the expression patterns of biosynthesis related genes and biological activity in Chinese cabbage (*Brassica rapa* spp. *pekinensis*). Sci Hortic.

[CR78] Trong TT, Truong DH, Nguyen HC, Tran DT, Thi HTN, Do Dang G, Huu HN (2017). Biomass accumulation of *Panax vietnamensis* in cell suspension cultures varies with addition of plant growth regulators and organic additives. Asian Pac J Trop Med.

[CR79] Tshabalala BD, Alayande KA, Sabiu S, Ashafa AOT (2016). Antimicrobial and anthelmintic potential of root and leaf extracts of *Gazania krebsiana* Less. Subsp. *serrulata* (DC.) Roessler: an in vitro assessment. Eur J Integr Med.

[CR80] Van Beek TA, Maas P, King BM, Leclercq E, Voragen AGJ, De Groot A (1990). Bitter sesquiterpene lactones from chicory roots. J Agric Food Chem.

[CR81] Veerashree V, Anuradha CM, Kumar V (2012). Elicitor-enhanced production of gymnemic acid in cell suspension cultures of *Gymnema sylvestre* R. Br Plant Cell Tiss Organ Cult.

[CR82] Wang W (2013) Studies on sexual reproduction biology and progenies phenotype analysis of *Gazania rigens* L. Dissertation, Soochow University, Suzhou (**in Chinese**)

[CR83] Wang H, Ma C, Li Z, Ma L, Wang H, Ye H, Xu G, Liu B (2009). Effects of exogenous methyl jasmonate on artemisinin biosynthesis and secondary metabolites in *Artemisia annua* L. Ind Crop Prod.

[CR84] Wiktorowska E, Długosz M, Janiszowska W (2010). Significant enhancement of oleanolic acid accumulation by biotic elicitors in cell suspension cultures of *Calendula officinalis* L. Enzyme Microb Technol.

[CR85] Xie LM, Hu JX, Huang WC (2013). Discussion on cutting and rapid propagation technology of *Gazania rigens* L. Jiangsu Agric Sci.

[CR86] Złotek U (2017). Effect of jasmonic acid and yeast extract elicitation on low-molecular antioxidants and antioxidant activity of marjoram (*Origanum majorana* L.). Acta Sci Pol Technol Aliment.

[CR87] Złotek U, Michalak-Majewska M, Szymanowska U (2016). Effect of jasmonic acid elicitation on the yield, chemical composition, and antioxidant and anti-inflammatory properties of essential oil of lettuce leaf basil (*Ocimum basilicum* L.). Food Chem.

